# The Last Wonder of the Spectroscope

**Published:** 1869-05

**Authors:** 


The Last Wonder of the Spectroscope.—The spectroscope, which, since its invention eight years since, by Bunsen and Kirchoff, has contributed so much to the progress of science, was used with signal success in observations of the recent total eclipse of the sun, by English and French parties, in different parts of Asia. By this means the nature of the protuberances on the rim of the solar disc, observed in former eclipses, has been satisfactorily explained. They are found to be columns of incandescent gas, possibly containing hydrogen.

